# wtest: an integrated R package for genetic epistasis testing

**DOI:** 10.1186/s12920-019-0638-9

**Published:** 2019-12-24

**Authors:** Rui Sun, Xiaoxuan Xia, Ka Chun Chong, Benny Chung-Ying Zee, William Ka Kei Wu, Maggie Haitian Wang

**Affiliations:** 1 Division of Biostatistics and Centre for Clinical Research and Biostatistics(CCRB), JC School of Public Health and Primary Care, the Chinese University of Hong Kong, Sha Tin, Hong Kong SAR, China; 2 Centre for Clinical Trials and Biostatistics, CUHK Shenzhen Research Institute, Shenzhen, China; 3 Institute of Digestive Diseases and Department of Medicine & Therapeutics, State Key Laboratory of Digestive Diseases, LKS Institute of Health Sciences, CUHK Shenzhen Research Institute, Shenzhen, China; 4 Department of Anesthesia, the Chinese University of Hong Kong, Sha Tin, Hong Kong SAR, China

**Keywords:** Epistasis testing, Association study, R package

## Abstract

**Background:**

With the increasing amount of high-throughput genomic sequencing data, there is a growing demand for a robust and flexible tool to perform interaction analysis. The identification of SNP-SNP, SNP-CpG, and higher order interactions helps explain the genetic etiology of human diseases, yet genome-wide analysis for interactions has been very challenging, due to the computational burden and a lack of statistical power in most datasets.

**Results:**

The wtest R package performs association testing for main effects, pairwise and high order interactions in genome-wide association study data, and cis-regulation of SNP and CpG sites in genome-wide and epigenome-wide data. The software includes a number of post-test diagnostic and analysis functions and offers an integrated toolset for genetic epistasis testing.

**Conclusions:**

The wtest is an efficient and powerful statistical tool for integrated genetic epistasis testing. The package is available in CRAN: https://CRAN.R-project.org/package=wtest.

## Background

The etiology of complex disorder involves an interplay of polygenic biomarkers, lifestyle and environmental factors [1]. Robust and efficient statistical tools are needed to perform interaction analysis in high volume genome data. Besides SNP-SNP interactions, the analysis of interactions of SNPs and cytosine-phosphate-guanine (CpG) sites might provide novel insight into the regulatory mechanism DNA methylation and gene expression underlying complex diseases.

Here we introduce a software that provides estimations for different types of genetic associations, including the main effect, second or higher order interaction, and gene-methylation interaction. This package is built upon the W-test [2] to perform epistasis testing. The statistic compares distributional differences of a set of biomarkers in cases and controls and follows a chi-squared distribution with data-set adaptive degrees of freedom. The method has the advantage of correcting *p*-value bias caused by complicated genetic architectures. Flexible implementation options are provided. The package can calculate SNP-CpG epistasis for biomarkers located in physical proximity of the input genome and epigenome. A number of post-test diagnostic, visualization and statistical genetic analysis functions are provided for model diagnosis. This is the first statistical software providing functions for direct gene-methylation interaction and high-order interaction evaluations in genome and epigenome dataset.

## Implementation

### Design

The wtest package is based on the W-test [2] to measure the association between binary phenotype and categorical genetic data. To test the association of a subset marker, a *k* by 2 contingency table can be formed, where *k* is the number of non-empty category combination formed by the SNP-set, and 2 is the binary phenotype. The statistic tests for the existence of distributional difference of a subset in the case group from a comparison control group, and it takes the following form,

1 $$  W=h\sum_{i=1}^{k}\left[\log{\frac{\hat{p}_{1i}/(1-\hat{p}_{1i})}{\hat{p}_{0i}/(1-\hat{p}_{0i})}/SE_{i}}\right]^{2} \sim\chi_{f}^{2}  $$

where *n*_1*i*_ and *n*_0*i*_ are the number of cases and controls in the *i*^*th*^ cell of the contingency table; *N*_1_ and *N*_0_ are the total cell counts of cases and controls; $\hat {p}_{1i} = n_{1i}/N_{1}$ and $\hat {p}_{0i} = n_{0i}/N_{0}$ are the conditional cell probabilities of the *i*^*th*^ cell of the contingency table; and *SE*_*i*_ is the standard error of the *i*^*th*^ log odds ratio. The W-test follows a chi-squared distribution of *f* degrees of freedom. The scalar *h* and degree of freedom *f* take forms of covariance matrices of the log odds ratios and are estimated from bootstrapped samples under the null hypothesis by the large sample theory. The W-test inherits a data-set adaptive degree of freedom that absorbs the genetic variation not attribute to phenotypes, therefore robust to complicated genetic architectures. In this software, we further extend it to evaluate high-order interaction effect and gene-methylation interaction effect. For gene-methylation interaction, methylation data are clustered into two categories according to high and low methylation levels by two-mean clustering algorithm. We also use a novel triangular network diagram to display interaction effects up to the third order. Extensive simulation studies testing the power and type I error of the W-test can be found in Wang, Sun et al. (2016) [2] and Sun et al. (2017) [3].

### Implementation

**Fig. 1:**
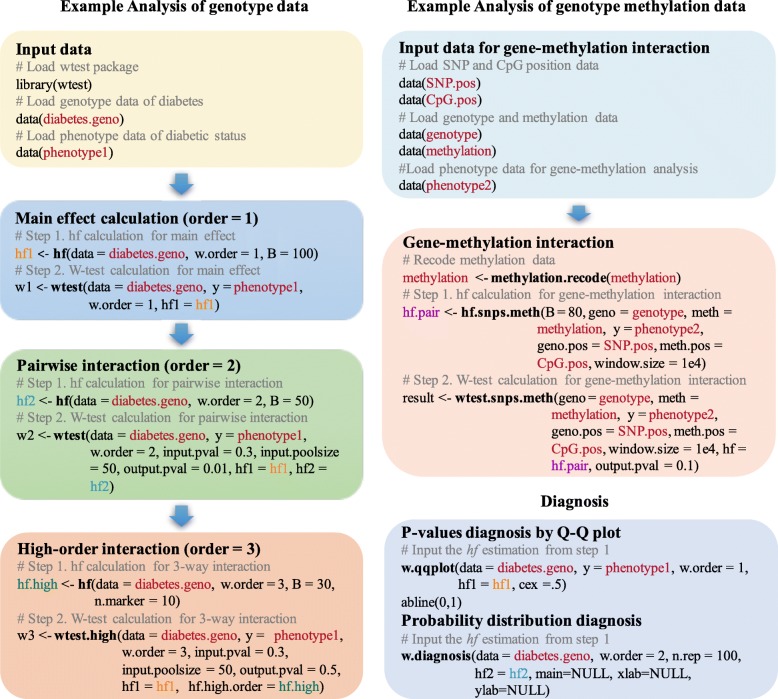
Integrated genetic epistasis testing and functions

Figure 1 demonstrates the major functions in the package and illustrates the implementation step by step using example data in the package. The implementation is performed in two steps: (1) Estimation of parameters *h* and *f*; (2) Testing by the W-test. Step 1. Estimation of parameters *h* and *f*. In genotype data, the *hf()* function is called, and in genotype and methylation data, the function *hf.snps.meth()* is called. Parameter *h* is the scaler in Eq. (1) and *f* is the degrees of freedom of a chi-squared distribution of the W-test. The two parameters are estimated using bootstrap samples with permutated phenotypes (null hypothesis) for B times. Simulations suggest that the estimation converges at *B*>400 when the number of variables is 1000 and the number of subjects is 1000 (Additional file 1). If step 1 is not performed, the *p*-value of W-test will be calculated by default *h* and *f*: *h*=*k*/(*k*−1) and *f*=*k*−1. In this case, *k* is the integer categorical combinations formed by the marker set. When *k*=2, the W-test is equivalent to the odds ratio test for a 2-by-2 table.

Step 2. Testing by the W-test. The *wtest()* evaluates main and second order interaction and *wtest.high()* evaluates third or higher order interaction in genotype data. The *wtest.snps.meth()* calculates SNP-CpG interactions for genome and epigenome data. Oftentimes users are interested to explore the interactions among biomarkers with a certain level of main effect signals. The *input.pval* option in the function can be used to screen candidate SNPs according to their *p*-values to form interaction sets. While the *output.pval* option allows the convenient output of interaction sets reaching a *p*-value threshold. In function *wtest.snps.meth()*, positions of the biomarkers are input alongside the genome and epigenome data sets, and the window size to calculate cis-regulation relationship can be specified. The *methylation.recode()* function transforms the methylation data into high and low methylated levels. For high order interaction calculation, a simple check for sample size can be done by estimating the average number of cell counts formed by a set, and a high order is feasible if the number is at least two. A reference table could be found in Additional file 2 with suggested sample sizes for various order of interactions.

Diagnostic checking for test statistic distribution can be performed by *w.diagnosis()*, which plots the W-test statistics histograms from the observed data and the curve of the chi-squared distribution using estimated parameters, indexed by the number of categorical combinations *k*. Close overlaying of the densities indicates the goodness of fit of estimation. An example is shown in the real data application section. The *w.qqplot()* function assists the diagnostic of probability distribution and degree of population stratification.

## Results

### Real data example

The software is applied to a number of real data analysis with novel biomarker findings and interesting implications [2–9]. Here we demonstrate its usage by two data sets: a genotypic dataset for bipolar disorder from the Genetic Association Information Network (GAIN) project, and a gene-methylation data for the lipid control treatment.

#### *Application I. GAIN bipolar disorder dataset*

**Fig. 2:**
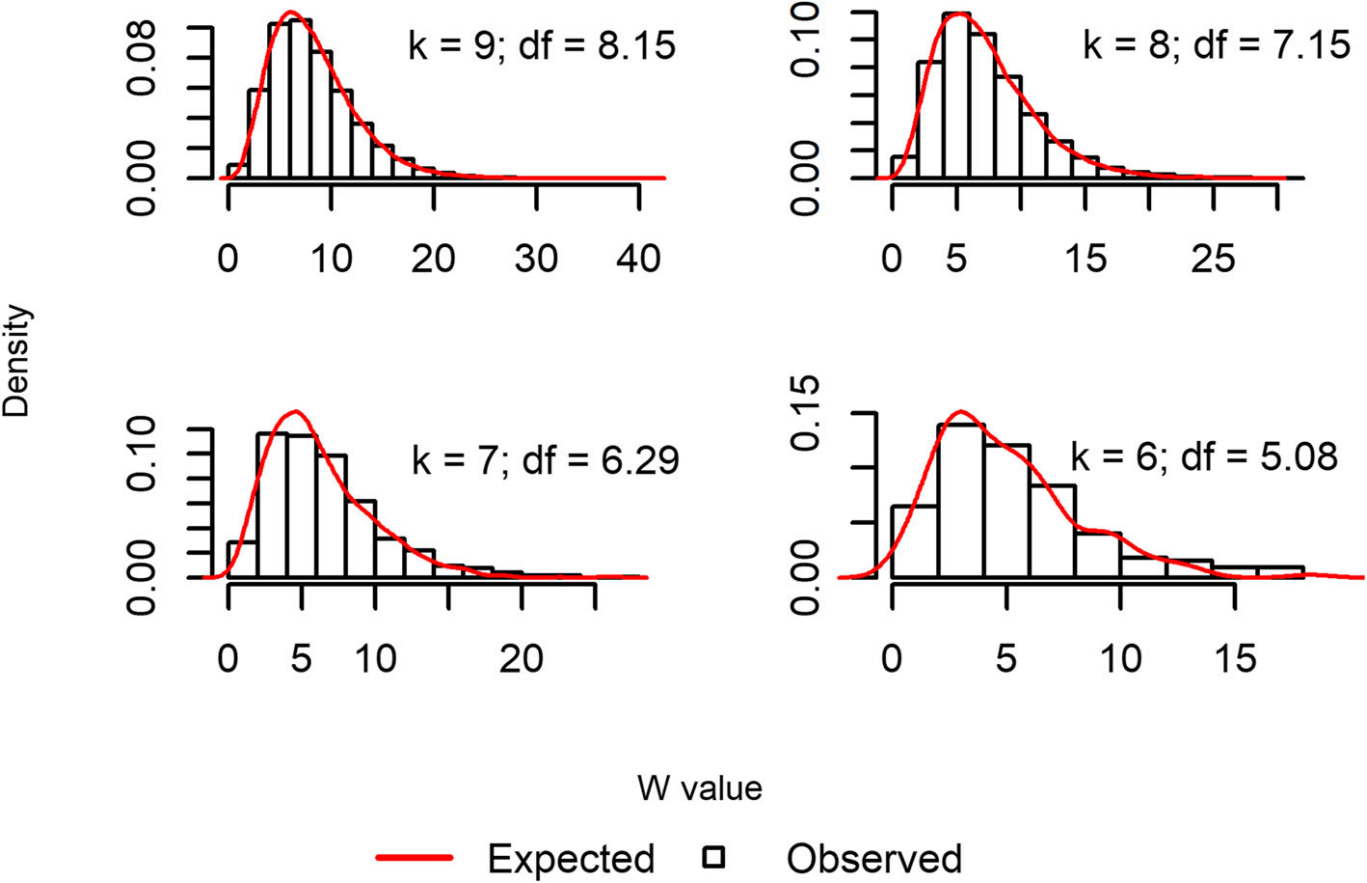
Diagnostic plot by *w.diagnostics*. At each combination size *k*, the estimated red color chi-square curve follows closely with the histogram of the W-test statistics calculated from the observed data, showing a good estimation of the parameters

This data contains 653 bipolar disorder patients and 1767 healthy controls, and 46,181 SNPs of chromosome 6 [10]. The result of *h* and *f* estimation can be found in Additional file 3. At second order interaction (*order*=2), setting *input*.*pval*=0.001 and *output*.*pval*=0.001, the function would output second order epistasis marker pairs with *p*-value <0.001. Figure 2 is the diagnostic plot for this estimation using *w.diagnosis()* function. The estimated red color chi-square curve follows closely with the histogram of the test statistics calculated from the observed data, showing a good estimation of the parameters.

**Fig. 3:**
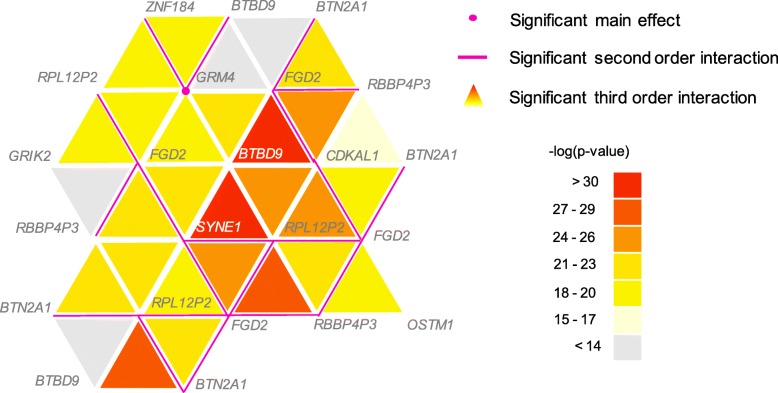
Triangular network for third order genetic interactions

Data analysis identified one SNP with significant main effect: rs2495982 near *GRM4*, *p*-value =2.06×10^−7^. *GRM4* is a major excitatory neurotransmitter in central nervous system and it is a susceptible gene for bipolar disorder and schizophrenia [11, 12]. For interaction effects, a number of SNP sets surpassed the Bonferroni corrected significance level. The top SNPs identified from different orders of interaction are listed in Additional file 4, and the interaction network up to the third order is plotted in a triangular network in Fig. 3. Each colored triangle in the network indicates a significant third order interaction, and the bold edge shows a significant second order interaction. It could be seen from the plot that the strongest interaction is formed by the gene set (*SYNE1*, *BTBD9*, *RPL12P2*) in the middle of the plot, in which *BTBD9* plays a key role and extends to form significant combinations with *FGD2* and *CDKAL1*. The *BTBD9* is reported to be associated with neuropsychiatric disorders such as restless legs syndrome in Schizophrenia and the Tourette Syndrome [13, 14]. The gene encodes the *BTB/POZ* domain-containing protein that involved in protein-protein interactions [15], and is highly expressed in brain tissues [16]. It is very encouraging to discover this gene with known physical protein interaction function from pure computational and statistical perspective.

#### *Application II. gene-methylation interaction analysis for lipid control data*

This application was originally reported in Sun *et al* 2018 [3]. The data set contains 476 diabetic patients undergone lipid control treatments, and 150,000 candidate SNP-CpG pairs within 10kb genome distance (*window*.*size*=10,000). The phenotype is whether or not a subject responded to the treatment, calculated by comparing the before and after treatment triglyceride levels [3]. The *h* and *f* are estimated by *hf.snps.meth()*, and the gene-methylation interactions are calculated by *wtest.snps.meth()*. Table 1 summarized the top 5 markers identified by gene-methylation interaction associations. The cluster of genes is found to be involved in neuronal and retinal functions, including *MPPED2* [17] and *GUCY2E* [18].

**Table 1:** Gene-methylation interaction in lipid control data

	SNP	CpG	Distance(kb)	Gene	MAF	*P*-value
1	rs12288568	cg13342435	1.27	*MPPED2*	0.003	7.49×10^−6^
2	rs11031153	cg13342435	3.86	*MPPED2*	0.003	7.49×10^−6^
3	rs16921036	cg13342435	1.35	*MPPED2*	0.001	8.68×10^−6^
4	rs11237066	cg13340272	4.52	*GUCY2E*	0.120	1.57×10^−5^
5	rs7119411	cg17432267	3.75	*C11orf63*	0.430	1.65×10^−5^

### Performance

The speed of the wtest package is evaluated on a laptop computer of 1.6GHz Intel Core i5 processor and 4GB RAM. Simulation data are used to compare the speed of different methods. On a data set consists of 5000 subjects and 100 SNPs, when *B*=200, *n*.*sample*=1000, the time elapsed for estimating *h* and *f* is 40.5s. After *h* and *f* calculation or assuming default values, the time used to evaluate main effects is 0.04s, and took 1.69s for second order interaction. In the same environment, the running time for existing tests for interaction yields 36.41s by chi-squared test and 130.56s by logistic regression. In the real data set, the genome-wide main effect calculation on 5000 subjects and 500,000 SNPs took around 5 min; and second order interaction calculation on 8000 SNPs used around 3.5 h.

## Conclusions

Genetic epistasis testing is important to fathom the massive genomic data, and it also provides a way to explore the relationship between diseases and various types of biomarkers. This package offers an integrated toolset to analyse the association of genetic signals at all levels: from main effects, high order interactions, to gene-methylation interactions. The software is available in CRAN from https://CRAN.R-project.org/package=wtest under the GPL-2.0 license.

## Availability and requirements

**Project name:** wtest

**Project home page:** https://CRAN.R-project.org/package=wtest

**Operation systems:** Platform independent

**Programming language:** R (>= 3.1), C++

**License:** GPL (>= 2)

**Restrictions to use by non-academics:** None

## Supplementary information

**Additional file 1** Convergency simulation study. The coefficient of variance of *h* at different *B* for pairwise interactions. Simulated dataset contains 1000 subjects and 1000 SNPs. A convergent *h* and *f* estimation is reached at *B*>400.

**Additional file 2** Reference table of sample size estimation. When the averaged MAF is 0.3 and the sample size is greater than the estimated sample size, no more than 25% cells have averaged cell count less than 2 in the contingency tables.

**Additional file 3** *h* and *f* estimation for main effects, second order interaction, and third order interaction analysis.

**Additional file 4** Top three identified sNPs at different levels of interaction orders. Note: Bonferroni corrected significant thresholds: main effect *p*-value <1.1×10^−6^, second order interaction *p*-value <4.69×10^−11^, and third order interaction *p*-value <3.05×10^−15^. Gene: the gene located within 35kb of the identified SNPs.

## Data Availability

The data mentioned in figure 1 are provided in the wtest package at https://CRAN.R-project.org/package=wtest. Raw sequence data for application 1 and 2 are available via the referenced manuscripts.

## Abbreviations

CpG: cytosine-phosphate-guanine
GAIN: association information network
SNP: Single-nucleotide polymorphism

## References

[1] Hunter DJ (2005). Gene-environment interactions in human diseases. Nat Rev Genet.

[2] Wang MH, Sun R, Guo J, Weng H, Lee J, Hu I (2016). A fast and powerful W-test for pairwise epistasis testing. Nucleic Acids Res.

[3] Sun R, Weng H, Men R, Xia X, Chong KC, Wu WKK (2018). Gene-methylation epistatic analyses via the W-test identifies enriched signals of neuronal genes in patients undergoing lipid-control treatment. BMC Proc.

[4] Wang YM, Ma L, Lu SY, Chan TCY, Yam JCS, Tang SM (2018). Analysis of multiple genetic loci reveals MPDZ-NF1B rs1324183 as a putative genetic marker for keratoconus. Br J Ophthalmol.

[5] Wu WKK, Sun R, Zuo T, Tian Y, Zeng Z, Ho J (2018). A novel susceptibility locus in MST1 and gene-gene interaction network for Crohn’s disease in the Chinese population. J Cell Mol Med.

[6] Wang MH, Chang B, Sun R, Hu IC, Xia XX, Wu WKK (2017). Stratified polygenic risk prediction model with application to CAGI bipolar disorder sequencing data. Hum Mutat.

[7] Sun R, Weng HY, Hu IC, Guo JF, Wu WKK, Zee BCY (2016). A W-test collapsing method for rare-variant association testing in exome sequencing data. Genet Epidemiol.

[8] Wang MH, Weng H, Sun R, Lee J, Wu WKK, Chong KC (2017). A Zoom-Focus algorithm (ZFA) to locate the optimal testing region for rare variant association tests. Bioinformatics.

[9] Uppu S, Krishna A (2018). A deep hybrid model to detect multi-locus interacting SNPs in the presence of noise. Int J Med Inform.

[10] McInnis MG, Dick DM, Willour VL, Avramopoulos D, MacKinnon DF, Simpson SG (2003). Genome-wide scan and conditional analysis in bipolar disorder: Evidence for genomic interaction in the National Institute of Mental Health Genetics Initiative bipolar pedigrees. Biol Psychiat.

[11] Fallin MD, Lasseter VK, Avramopoulos D, Nicodemus KK, Wolyniec PS, McGrath JA (2005). Bipolar I disorder and schizophrenia: A 440-single-nucleotide polymorphism screen of 64 candidate genes among Ashkenazi Jewish case-parent trios. Am J Hum Genet.

[12] Kato T (2007). Molecular genetics of bipolar disorder and depression. Psychiatr Clin Neurosci.

[13] Janik P, Berdynski M, Safranow K, Zekanowski C (2014). The BTBD9 gene polymorphisms in Polish patients with Gilles de la Tourette syndrome. Acta Neurobiol Exp (Wars).

[14] Guo Y, Su L, Zhang J, Lei J, Deng X, Xu H (2012). Analysis of the BTBD9 and HTR2C variants in Chinese Han patients with Tourette syndrome. Psychiatr Genet.

[15] Gene N. BTBD9 BTB domain containing 9 [Homo sapiens (human)]. 2018. https://www.ncbi.nlm.nih.gov/gene/114781#gene-expression. Accessed 1 Nov 2018.

[16] Fagerberg L, Hallstrom BM, Oksvold P, Kampf C, Djureinovic D, Odeberg J (2014). Analysis of the human tissue-specific expression by genome-wide integration of transcriptomics and antibody-based proteomics. Mol Cell Proteomics.

[17] Gormley P, Anttila V, Winsvold BS, Palta P, Esko T, Pers TH (2016). Meta-analysis of 375,000 individuals identifies 38 susceptibility loci for migraine. Nat Genet.

[18] Perrault I, Rozet JM, Calvas P, Gerber S, Camuzat A, Dollfus H (1996). Retinal-specific guanylate cyclase gene mutations in Leber’s congenital amaurosis. Nat Genet.

