# Supplementary material for: wtest: an integrated R package for genetic epistasis testing
Source: BMC Med Genomics. 2019 Dec 24;12(Suppl 9):180. doi: 10.1186/s12920-019-0638-9 (PMC6929460; doi:10.1186/s12920-019-0638-9)

Additional file 1 – Convergency simulation study
Coefficient of variance of *h* at different B for pairwise interactions. Simulated dataset contains 1,000 subjects and 1,000 SNPs. A convergent *h* and *f* estimation is reached at B > 400.

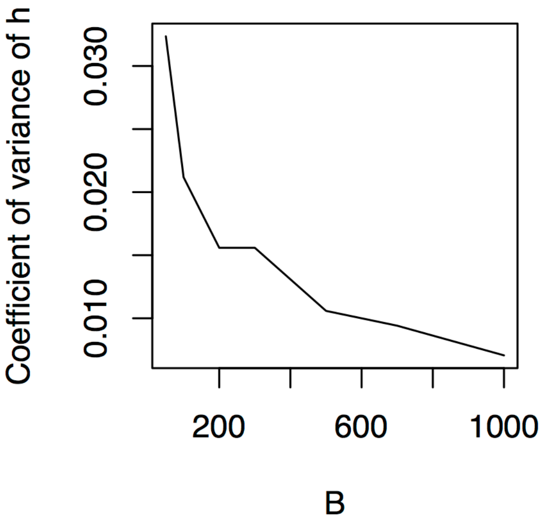

Supplement: Supplementary file 1 — Additional file 1 Convergency simulation study. The coefficient of variance of h at different B for pairwise interactions. Simulated dataset contains 1000 subjects and 1000 SNPs. A convergent h and f estimation is reached at B>400. [file 12920_2019_638_MOESM1_ESM.pdf]
